# Tracheoesophageal Fistula in a COVID-19 Ventilated Patient: A Challenging Therapeutic Decision

**DOI:** 10.1155/2021/6645518

**Published:** 2021-03-30

**Authors:** Riccardo Rosati, Paola De Nardi, Antonio Dell'Acqua, Maria Rosa Calvi, Ugo Elmore, Elena Scarparo, Luigi Beretta

**Affiliations:** ^1^Gastrointestinal Surgery, IRCCS San Raffaele Scientific Institute, Milan, Italy; ^2^School of Medicine, Vita-Salute San Raffaele University, Milan, Italy; ^3^Department of Anesthesia and Intensive Care, IRCCS San Raffaele Scientific Institute Milan, Italy

## Abstract

COVID-19 associated severe respiratory failure frequently requires admission to an intensive care unit, tracheal intubation, and mechanical ventilation. Among the risks of prolonged mechanical ventilation under these conditions, there is the development of tracheoesophageal fistula. We describe a case of a severe COVID-19 associated respiratory failure, who developed a tracheoesophageal fistula. We hypothesized that one of the mechanisms for tracheoesophageal fistula, along with other local and general risk factors, is the local infection due to the location of the virus itself in the tracheobronchial tree. The patient was managed successfully with surgical intervention. This case highlights the increased risk of this potentially life-threatening complication among the COVID-19 patient cohort and suggests a management strategy.

## 1. Background

The COVID-19 pandemic caused by the SARS-CoV-2 virus has dramatically and rapidly spread around the world in recent months. A wide spectrum of clinical manifestations with different degrees of severity may occur, including those associated with the virus' predominant symptom of severe respiratory distress.

Patients who require intensive care for respiratory failure are critical due to the severity of the illness and to the risks associated with prolonged mechanical ventilation and intensive care unit (ICU) stay; among them, pulmonary barotrauma due to positive ventilation and high positive end-expiratory pressure (PEEP) has to be considered [1]. In addition, in patients with COVID-19 infection, the direct insult of the virus on the upper airway cells, causing inflammatory infiltrate on the mucosa and submucosa, can further weaken the structure of the trachea [2]. This might possibly result either in perforation or in a fistula between the posterior wall of the trachea and the anterior wall of the esophagus, compressed between the orotracheal tube cuff and the nasogastric tube.

We present the case of a patient with severe COVID-19-associated pneumonia who underwent orotracheal intubation and prolonged mechanical ventilation. The patient developed a tracheoesophageal fistula (TEF) successfully repaired. The decisional algorithm should balance between an early effective surgical repair and the need of maintaining ventilation for a long-lasting respiratory failure. Different therapeutic options are available and discussed.

## 2. Case Presentation

On March 13, 2020, a 52-year-old man with obesity (BMI = 34) and with new-onset diabetes was admitted for fever and cough for six days. He tested positive for SARS- CoV-2 virus. After 2 days, he developed increasing dyspnea, fatigue, and decreasing oxygen saturation despite treatment with noninvasive ventilation (NIV), and he was transferred to the ICU. Intensive respiratory treatments was started including prone position, 20% of the day on average during the first week, due to obesity of the patient and high risk of developing pressure sores. The patient was placed on pressure-regulated volume control with the following settings: tidal volume 500 mL; respiratory rate 24; inspiratory/expiratory ratio 1/1,3; static compliance: 24; PEEPi: 0. Nevertheless, the PaO2/FiO2 ratio was <120.

**Figure 1:**
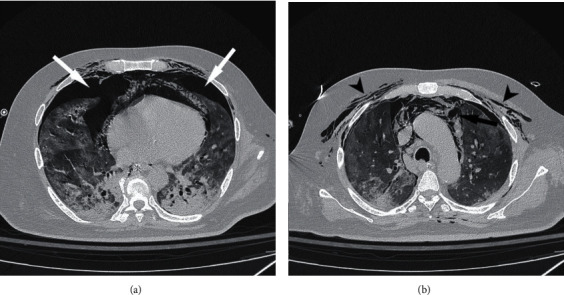
Chest CT. A chest CT scan shows bilateral pneumothorax (white arrows) (a), subcutaneous cervical and thoracic emphysema (arrowheads), and pneumomediastinum (black arrow) (b). Bilateral, patchy ground-glass opacities consistent with interstitial pneumonia are also shown.

**Figure 2:**
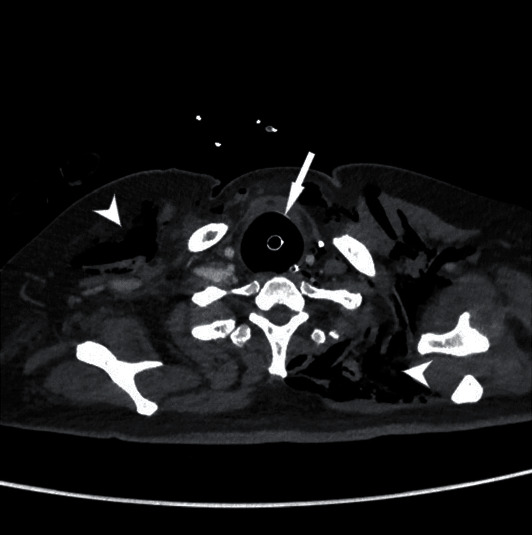
Neck CT. An overinflated tracheal tube cuff and dilated trachea can be seen compressing the cervical esophagus.

On hospital day 25, the patient developed thoracic subcutaneous emphysema and increased troponin-T and NT-proBNP levels. A chest X-ray showed bilateral pneumothorax and subcutaneous emphysema. A chest CT scan confirmed the radiological findings and revealed the presence of a large pneumomediastinum (Figures 1 and 2) and an overinflated tracheal tube cuff (maximum diameter: 4 cm); the cuff pressure was 20 cmH20. Both pleural cavities were drained. On hospital day 28, massive subcutaneous bilateral emphysema in the cervical and anterior thoracic region and massive air inflation into the nasogastric tube occurred. A chest X-ray revealed overdistension of the stomach. A tracheoesophageal fistula (TEF) was thus suspected. Bronchoscopy confirmed a TEF of approximately 2 cm located 3 cm distal to the vocal cords and 5 cm proximal to the carina and an enlarged trachea lumen. Under direct bronchoscopy, the endotracheal tube was placed distal to the TEF. Surgery was scheduled to repair the tracheal and esophageal tears while performing a tracheostomy. On hospital day 30, the patient underwent surgery. First, an upper GI endoscopy was performed in order to properly localize the esophageal fistula, which was in the anterior esophageal wall approximately 4 cm distal to the upper esophageal sphincter (UES). A percutaneous endoscopic gastrostomy was fashioned for stomach decompression and enteral feeding. A left cervicotomy was then performed at the border of the sternocleidomastoid muscle, and the cervical esophagus was carefully dissected from the UES to the thoracic inlet. The laryngotracheal block was also mobilized and dissected. A fistula approximately 4 cm long was identified. The trachea and esophagus were separated, and a sharp debridement of the fistulas margins was carried out. The membranous wall of the trachea was sutured with simple interrupted stitches of 4-0 absorbable monofilament. Then, the esophagus was repaired in two layers using the same material. The esophageal suture tightness was assessed using an underwater pneumatic test via the nasogastric tube. A pedicled muscular flap was prepared using the left sternocleidomastoid muscle, which was passed around the esophageal suture as a scarf to separate the tracheal plane. The flap was fixed in position with interrupted sutures to the trachea and esophagus. A tracheostomy was finally performed with the cannula cuff placed distal to the tracheal repair.

**Figure 3:**
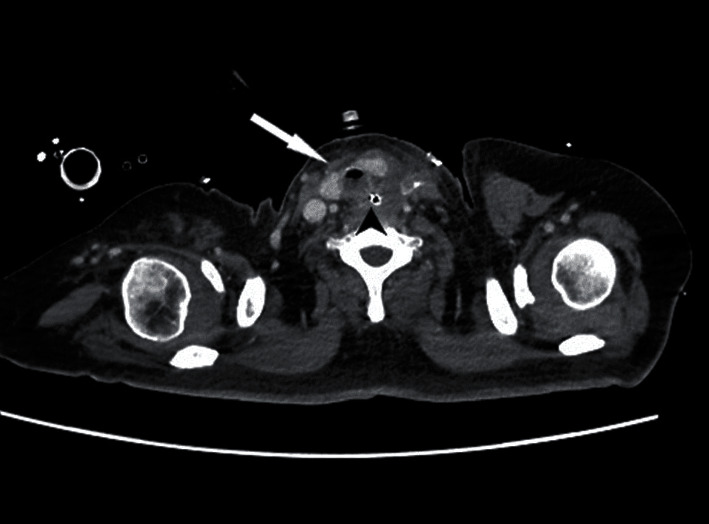
Postoperative neck CT. A neck CT obtained on postoperative day 10 shows no free air between the trachea (arrow) and the esophagus (arrowhead) at the level of the tracheoesophageal fistula repair (above the tracheostomy).

The repair was checked on postoperative day 10 via a CT scan (Figure 3) and on postoperative day 15 via endoscopy. The postoperative course was complicated by recurrent laryngeal nerve palsy and tracheal stenosis, not involving the site of repair but the whole circumferentia of the lumen. The stenosis was treated with placement of a Montgomery T tube. The patient was discharged from the ICU 15 days after surgery, on a regular oral diet. Speech therapy rehabilitation was scheduled. The patient is now well 8 months after surgery, the percutaneous gastrostomy was removed 3 months after the operation, and the patient resumed a normal diet with regain of his usual weight; at the last bronchoscopy, the stenosis was resolved and thus the Montgomery T tube was removed.

## 3. Discussion and Conclusions

Acquired, nonmalignant TEF is one of the fearful complications of prolonged intubation for mechanical ventilation. Several factors could facilitate its development such as cuff overinflation of the tracheal or tracheostomy tube [3], high airway pressure, excessive motion of the endotracheal tube, respiratory infections, presence of a nasogastric tube, esophageal infection, hypotension, old age, female gender, and concomitant immunosuppression or diabetes [4]. Multiple individual and general factors, such as obesity, diabetes, hemodynamic instability due to sedation, prolonged intubation, the need to change position (prone-supine) frequently with possible excessive motion of the tube, repeated bronchoscopies, and the need for high PEEP ventilation over a 4 weeks period, all possibly contributed to the development of the TEF. In our case, as reported for COVID-19 patients, the airway inflammatory process and edema could have weakened the structure of the tracheal wall [5] leading to collapse, tracheomalacia, and eventually to necrosis of the mucosa and to a fistula with the esophagus [6], even if routine cuff pressure measurements were performed and intracuff pressures would be always maintained lower than 20 cm H_2_O.

A recent consensus statement suggested to perform tracheostomy in COVID-19 patients expected to require prolonged intubation; however, a specific timing was not recommended [7]. Timing for performing a tracheostomy is still a matter of debate as several guidelines proposed early fashioning, while other guidelines suggest to wait for at least 14 days [8]. Earlier tracheostomy although probably desirable was deemed contraindicated in our patient due to severe hypoxia and high PEEP requirement.

The acute presentation of TEF in this patient is typical in patients undergoing mechanical ventilation, with a drop in oxygen saturation (in spite of adequate ventilation), positive cuff leaks, and gastric distension. The diagnosis of TEF is confirmed with a CT scan, endoscopy, or barium esophagography [9].

As the spontaneous closure of a TEF is rare, surgical repair is generally required. However, the timing of fistula closure in ventilator-dependent patients is a matter of debate. The majority of the authors advocate to postpone the repair until the patient is fully stable and weaned from mechanical ventilation, to reduce the risk of anastomotic dehiscence [10]. We considered early surgical repair in our patient since he was still critically ill and prolonged mechanical ventilation was expected. Delay in surgery could worsen ischemic damage and necrosis of the tracheal wall and enlarge an already 4 cm defect. This situation could challenge any further attempt of surgical management.

Our surgical approach was through a left lateral cervicotomy. Although either a transverse anterior collar or a U-shaped cervical incision might be preferred by some authors [11], a left lateral cervicotomy may also be used when, as in the present case, an esophageal involvement is present, and direct closure of both the fistulas is planned. When performing a primary repair, muscle interposition with a pedicled flap to separate the esophagus and the trachea suture lines is preferable. Several muscle flaps can be used [11]. The pectoralis major is generally preferred for better results due to its stronger vascular pedicle. In the present case, we preferred the left sternocleidomastoid muscle because it is simpler to prepare from the lateral cervical incision. Moreover, it allows for proper ventilation with no risk of graft compression in the prone position that could be convenient in COVID-19 ventilated patients.

Despite high-related morbidity and mortality of TEF, even higher in patients with COVID-19 [12], surgical repair must be contemplated. Because of a probable prolonged ventilation period needed for COVID-19 related respiratory failure, we preferred to plan surgical repair without further delay. The patient was then managed locally by placing the cuff of the tracheostomy cannula distally to the tracheal suture with low inflating pressure, thus, preserving the integrity of the repair.

As described, a tracheal stenosis was observed in the postoperative period. We hypothesized that the combination of tracheostomy and infection led to the formation of secondary scar tissue healing with shrinkage of the. The trachea stenosis was treated with the temporary placement of a Montgomery T-Tube. A vocal rehabilitation program was required due to temporary palsy of the vocal cords that, however, did not impair swallowing and the resuming of oral intake.

In summary, a COVID-19 patient with respiratory failure developed a precocious TEF. Due to an expected long-term respiratory dysfunction, immediate surgical repair was carried out, prior to weaning from mechanical ventilation. Repair of TEF was successful without life-threatening complications or major surgical sequelae. Surgical repair of TEF in COVID-19 patients is feasible and should probably be done earlier than later.

## Data Availability

Data can be requested to the authors.
